# Supplementary material for: Functional Assessment of 12 Rare Allelic CYP2C9 Variants Identified in a Population of 4773 Japanese Individuals
Source: J Pers Med. 2021 Feb 2;11(2):94. doi: 10.3390/jpm11020094 (PMC7912942; doi:10.3390/jpm11020094)
Supplement: Supplementary file 1 [file jpm-11-00094-s001.pdf]

Supplementary Materials

Functional assessment of 12 rare allelic *CYP2C9* variants identified in a population of 4,773 Japanese individuals

Masaki Kumondai, Akio Ito, Evelyn Marie Gutiérrez Rico, Eiji Hishinuma, Akiko Ueda, Sakae Saito, Tomoki Nakayoshi, Akifumi Oda, Shu Tadaka, Kengo Kinoshita, Masamitsu Maekawa, Nariyasu Mano, Noriyasu Hirasawa, Masahiro Hiratsuka

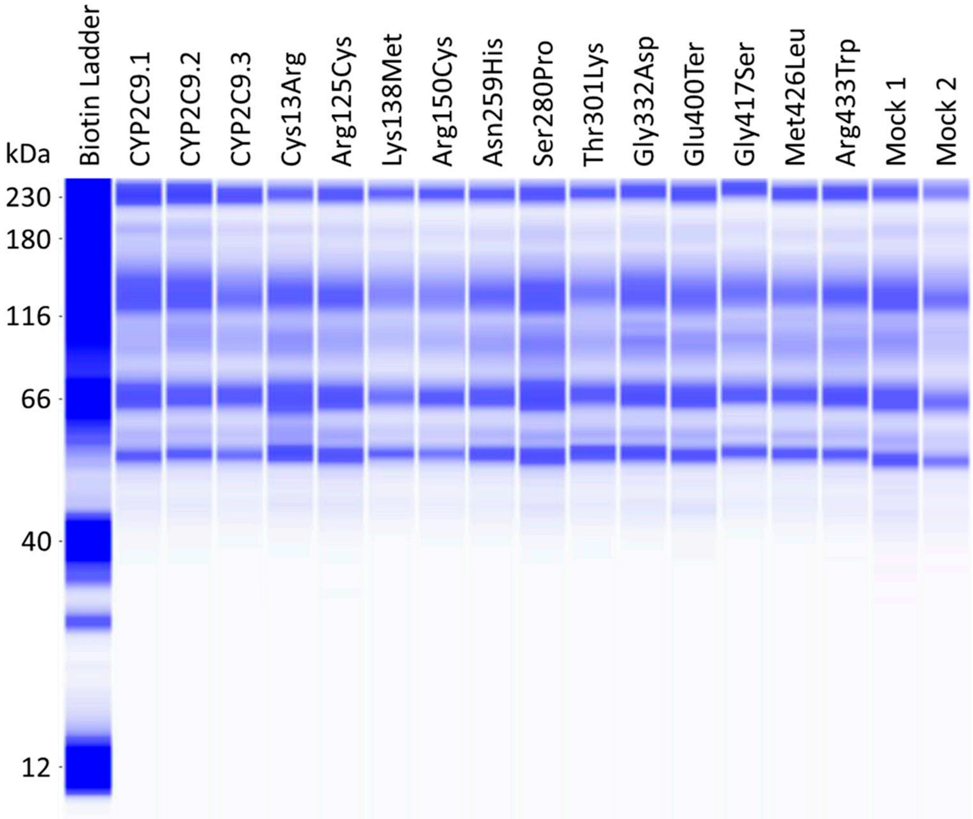

Figure S1. Representative western blots showing total proteins. Ter represents amino acid termination. Mock 1 indicates transfection with 10 μg mock plasmid. Mock 2 indicates transfection with 9.6 μg mock plasmid, 0.2 μg CPR plasmid, and 0.2 μg cytochrome b_5 plasmid. N.D. represents not determined.

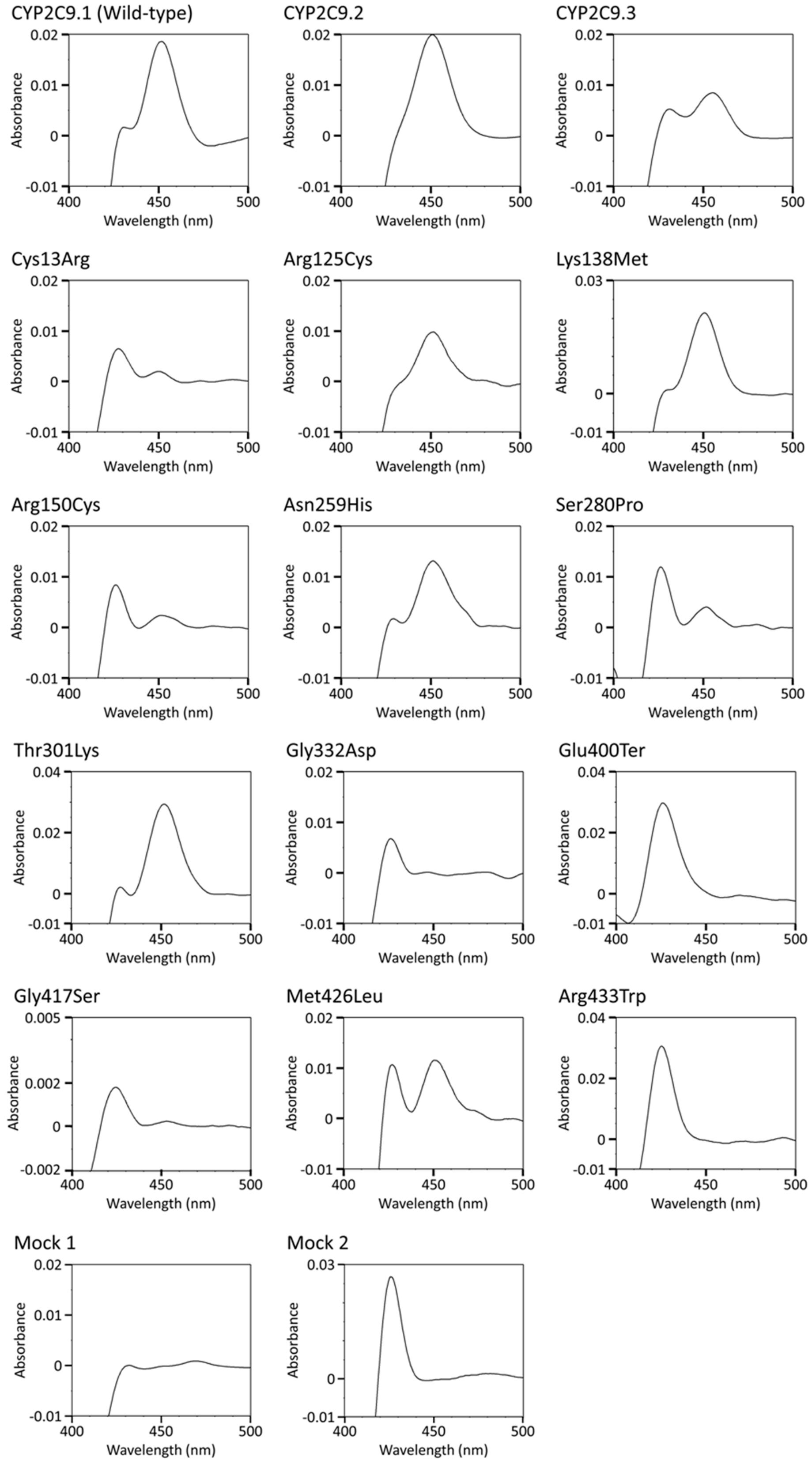

Figure S2. Representative CO-difference spectra of CYP2C9 variant proteins expressed in 293FT cells. All assays and measurements were performed in triplicate using a single microsomal preparation. Ter represents amino acid termination. Mock 1 represents transfection with 10 μg mock plasmid. Mock 2 represents transfection with 9.6 μg mock plasmid, 0.2 μg CPR plasmid, and 0.2 μg cytochrome b_5 plasmid.

Table S1. PCR primers used to amplify sequences of the human *CYP2C9* gene.

Exon	Size (bp)	Forward primer (5′-3′)	Reverse primer (5′-3′)
1	486	ATTTATCCATCAAAGAGGCACA	ATCTAACATGCAAAGACCCAAA
2	583	ATTGTCTGACCATTGCCTTGA	GCCGGATCTCCTTCCATTT
3	611	TGAAACCCATAGTGGTGCTG	CACAAATATGTGCAAATTCCCT
4	621	TTTAACCAGCTAGGTTGTAATG	AGAAACAGGGCTTTGGAGTTT
5	789	TGCTGTCATCTACAAAACGTGA	TAGTTTATATTTCTGTGGGCTC
6	429	GTCTACAGCCTCTGCTATACAAGCAG	CAGATTACAGCTGGAAGCACAAAAG
7	595	GTGCATCTGTAACCATCCTCT	TAAGAGTAGCCAAACCAATCTT
8	642	AAGCTTGATGAAAAGGAGGAC	TCTCAATTTGGAAGAATTGGATT
9	653	ATTTTTCAGTTGCCTATACATCC	GATAAGTATGAGCATTATGTGAC

Table S2. Already known *CYP2C9* allelic variants identified in 4773 Japanese individuals.

Variants	Nucleotide mutations	rs number	Amino acid substitutions	Frequency (%)
*CYP2C9*3*	1075A>C	rs1057910	Ile359Leu	2.36
*CYP2C9*8*	449G>A	rs7900194	Arg150His	0.31
*CYP2C9*13*	269T>C	rs72558187	Leu90Pro	0.07
*CYP2C9*16*	895A>G	rs72558192	Thr299Ala	0.01
*CYP2C9*27*	449G>T	rs7900194	Arg150Leu	0.02
*CYP2C9*28*	641A>T		Gln214Leu	0.01
*CYP2C9*29*	835C>A	rs182132442	Pro279Thr	0.21
*CYP2C9*30*	1429G>A	rs781583846	Ala477Thr	0.21
*CYP2C9*34*	1004G>A	rs367826293	Arg335Gln	0.02
*CYP2C9*36*	1A>G	rs114071557	Met1Val	0.02
*CYP2C9*43*	371G>A	rs12414460	Arg124Gln	0.02
*CYP2C9*44*	389C>T	rs200965026	Thr130Met	0.02
*CYP2C9*47*	488C>T	rs774550549	Pro163Leu	0.07
*CYP2C9*56*	1159A>G	rs764211126	Ile387Val	0.01

Table S3. Kinetic parameters of (*S*)-warfarin 7-hydroxylation by microsomes from 293FT cells expressing wild-type and variant CYP2C9 proteins.

**Variants**	K_m **(μM)**	k_{cat} **(fmol/min/pmol CYP2C9 holoprotein)**	**Catalytic efficiency (k_{cat}/K_m) (% of wild-type)**
CYP2C9.1 (wild-type)	0.88 ± 0.06	83.04 ± 1.19	94.50 ± 5.98 (100.00)
CYP2C9.2 (Arg144Cys)	1.06 ± 0.05	43.77 ± 2.84**	41.20 ± 1.15* (43.59)
CYP2C9.3 (Ile359Leu)	2.87 ± 2.11	25.65 ± 3.07***	11.69 ± 5.58*** (12.37)
Cys13Arg	2.36 ± 0.30	9.75 ± 0.83***	4.17 ± 0.46* (4.41)
Arg125Cys	2.45 ± 0.53	9.11 ± 0.41***	3.86 ± 1.02** (4.09)
Lys138Met	1.52 ± 0.13	47.45 ± 1.26***	31.38 ± 2.78** (33.21)
Arg150Cys	1.01 ± 0.10	404.83 ± 16.95**	404.27 ± 27.55* (427.79)
Asn259His	0.79 ± 0.04	104.83 ± 4.47	131.54 ± 5.33* (139.19)
Ser280Pro	1.33 ± 0.24	102.89 ± 6.48	79.05 ± 15.65 (83.65)
Thr301Lys	N.D.	N.D.	N.D.
Gly332Asp	1.87 ± 0.27	132.17 ± 3.78**	71.33 ± 8.15 (75.48)
Glu400Ter	N.D.	N.D.	N.D.
Gly417Ser	0.78 ± 0.04	284.31 ± 13.45*	366.29 ± 18.45** (387.60)
Met426Leu	0.86 ± 0.02	139.98 ± 3.24***	162.71 ± 6.59*** (172.17)
Arg433Trp	N.D.	N.D.	N.D.

Data represent the means ± SDs of the three independently performed catalytic assays. *$P < 0.05$, **$P < 0.01$, and ***$P < 0.005$ compared with wild-type CYP2C9 by Dunnett T3 tests. Ter represents amino acid termination. N.D. represents not determined. All assays and measurements were performed in triplicate using a single microsomal preparation.

Table S4. Kinetic parameters of tolbutamide 4-hydroxylation by microsomes from 293FT cells expressing wild-type and variant CYP2C9 proteins.

Variants	K_m (μM)	k_{cat} (pmol/min/pmol CYP2C9 holoprotein)	Catalytic efficiency (k_{cat}/K_m) (% of wild-type)
CYP2C9.1 (wild-type)	53.20 ± 1.86	3.19 ± 0.19	59.93 ± 3.68 (100.00)
CYP2C9.2 (Arg144Cys)	72.71 ± 6.84	2.21 ± 0.17	30.49 ± 1.32* (50.88)
CYP2C9.3 (Ile359Leu)	176.31 ± 7.07**	0.71 ± 0.01*	4.02 ± 0.15* (6.70)
Cys13Arg	104.64 ± 15.72	0.33 ± 0.04**	3.22 ± 0.23* (5.38)
Arg125Cys	76.80 ± 4.17	0.48 ± 0.01*	6.29 ± 0.35* (10.49)
Lys138Met	68.98 ± 3.24	1.94 ± 0.08*	28.13 ± 2.00* (46.93)
Arg150Cys	70.41 ± 2.38*	15.07 ± 0.34***	214.43 ± 13.83* (357.79)
Asn259His	36.90 ± 2.55*	2.15 ± 0.08	58.39 ± 3.99 (97.43)
Ser280Pro	79.63 ± 10.54	4.69 ± 0.13*	59.87 ± 8.67 (99.90)
Thr301Lys	N.D.	N.D.	N.D.
Gly332Asp	112.62 ± 32.21	4.28 ± 0.96	39.20 ± 5.31 (65.40)
Glu400Ter	N.D.	N.D.	N.D.
Gly417Ser	77.82 ± 3.07*	10.53 ± 0.48***	135.71 ± 12.75 (226.43)
Met426Leu	52.25 ± 2.99	4.48 ± 0.26	85.83 ± 2.15* (143.22)
Arg433Trp	N.D.	N.D.	N.D.

Data represent the means ± SDs of the three independently performed catalytic assays. *$P < 0.05$, **$P < 0.01$, and ***$P < 0.005$ compared with wild-type CYP2C9 by Dunnett T3 tests. Ter represents amino acid termination. N.D. represents not determined. All assays and measurements were performed in triplicate using a single microsomal preparation.
